# Intracranial Hematoma After Ventriculoperitoneal Shunt Placement in a Patient With Factor V Deficiency: A Rare Case Report

**DOI:** 10.7759/cureus.37302

**Published:** 2023-04-08

**Authors:** Yassine Ait M'barek, Hajar Hamadi, Lamia Benantar, Elmehdi Hamidi, Khalid Aniba

**Affiliations:** 1 Neurological Surgery, Ibn Tofail Hospital, Mohammed VI University Hospital, Marrakech, MAR

**Keywords:** bleeding disorder, paraheamophilia, vp shunt, intracranial hematoma, factor v deficiency

## Abstract

Congenital factor V deficiency (FVD) is a rare bleeding disorder due to an inherited mutation. So far, there are no standard protocols for pre- and peri-operative management of patients with factor V deficiency. This poses a challenge for surgeons and requires a multidisciplinary approach. We present a case of a 60-year-old woman with factor V deficiency admitted to the neurosurgery department of Ibn Tofail Hospital for hydrocephalus requiring a ventriculoperitoneal shunt. Pre-operative management of the patients as well as outcome and follow-up are described and compared with relevant literature.

## Introduction

Factor V deficiency (FVD), also known as parahemophilia or Owren disease, is a rare bleeding disorder due to a homozygous or compound heterozygous mutation [1]. The exact incidence of this inherited disorder is unknown, but it is believed to be less than one in every million live births [2]. The clinical presentation can range from minor ecchymoses to life-threatening hemorrhage [1,3,4]. FVD can be categorized according to the factor V percentage in the plasma into mild (>10%), moderate (1-10%), and severe (<1%) [5].

Among patients with FVD, intracranial hemorrhage is extremely rare in adults and usually occurs in children [6-8]. The only available treatment option for this hematologic disorder is the administration of significant amounts of fresh frozen plasma (FFP) [8,9]. This condition can increase per operative bleeding risk during cranial surgery. Moreover, articles on perioperative management in patients with FVD are scant [8].

We describe the case of a 60-year-old patient with FVD diagnosed with hydrocephalus who underwent a ventriculoperitoneal shunt (VP shunt) complicated with an intracranial hematoma. We report clinical, radiological, and biological findings, as well as management before, during, and after surgery, with a detailed postoperative follow-up.

## Case presentation

The patient, a 60-year-old woman with a personal and family history of FVD (an older sibling with FVD), presents to our department of neurosurgery with symptoms of headache, double vision, nausea, vomiting, and dizziness evolving for several weeks. An in-depth history showed that she underwent celioscopic cholecystectomy eight months prior to admission with an uneventful follow-up; on the other hand, she had no significant history of mucosal or gastrointestinal tract bleeding. 

On physical examination, the patient was asthenic and could not sustain the upward position due to dizziness. Neurological examination showed the presence of cerebellar syndrome along with preserved motor and sensory functions.

**Figure 1:**
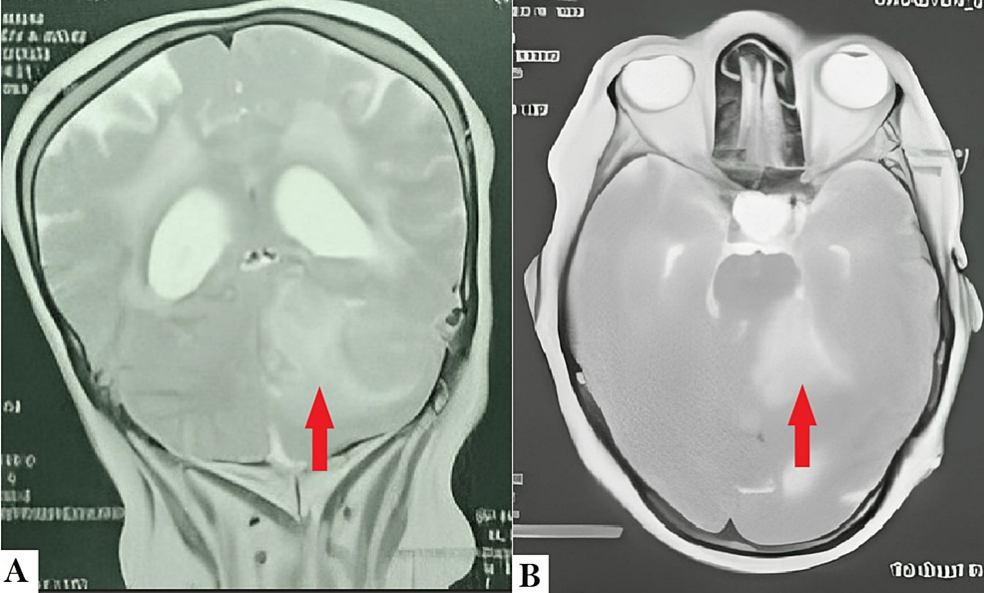
Cerebral MRI of the patient showing a left cerebellar pontine angle lesion responsible for obstructive tri-ventricular hydrocephalus. (A) Cerebral MRI in T2 sequence, coronal plane, showing a left cerebellar lesion with peripheral edema (red arrow) exerting a mass effect on the surrounding structures as well as obstructive hydrocephalus due to the compression of the fourth ventricle. (B) Cerebral MRI in flair sequence, axial plane, showing the same lesion centered on the left cerebellar pontine angle (red arrow) with important peripheral edema exerting a mass effect on the surrounding structures.

MR imaging of the brain showed the presence of a large compressive heterogenous mass located in the left cerebellar pontine angle, measuring 44×32.6×37.5 mm in hyposignal T1 and hypersignal T2. The mass was irregularly enhanced after gadolinium injection. Possible diagnoses at this point were meningioma, hemangiopericytoma, and less probably, a schwannoma. Moreover, the lesion causes obstructive tri-ventricular hydrocephalus and an important mass effect on the surrounding structures (Figure 1).

Further investigations were mandatory given the history of FVD. A full blood panel, as well as a clothing factors level test, was performed. The results showed hemoglobin and platelet rates within normal limits. Prothrombin level was 38%, international normalized ratio (INR) was at 1.5, and factor V level was at 12%. The rest of the clothing factor levels (especially factors II, VII, VIII, and X) were within the normal range (Table 1).

**Table 1:** Results of the patient's blood test results and a comparison with the normal range.

	Test result	Normal range
Hemoglobin	12.1 g /dl	12-16 g/dl
Platelet level	218,000/mm^3^	150,000-400,000/mm^3^
Prothrombin level	72%	70-100%
International normalized ratio (INR)	1.59	2-4
Factor II	108%	70-150%
Factor V	12%	62-150%
Factor VII	107%	67-143%
Factor X	92%	70-150%
Fibrinogen	3.58 g/L	2-4.6 g/L

After careful multidisciplinary discussion of the case problem with the anesthesiologists and hematologists teams, surgery of the intracranial process was deemed of extremely high bleeding risk and could be life-threatening despite preoperative and perioperative preparations and preventive measures. We decide that the placement of a VP shunt at this stage would be less risky and sufficient to improve the patient’s current complaints.

Prior to surgery, the patient received a bolus of 1100 ml of FFP (a recommended dose of 15 to 25 mL of FFP per kilogram of body weight). The control prothrombin level was 65% (INR 1.5), and factor V recovered to 25% (normal range from 62% to 150%) [2].

The patient underwent a VP shunt placement under general anesthesia. We did not experience any bleeding problems during surgery, and the hemostasis at the end of the procedure was satisfactory. Cerebrospinal fluid samples were collected for pathology and bacteriological examinations. She woke up with no neurological deficit and was admitted to the intensive care unit for a 24-hour observation period. The patient was then sent to the ward after an uneventful immediate postoperative follow-up and showed improvement in her symptoms.

**Figure 2:**
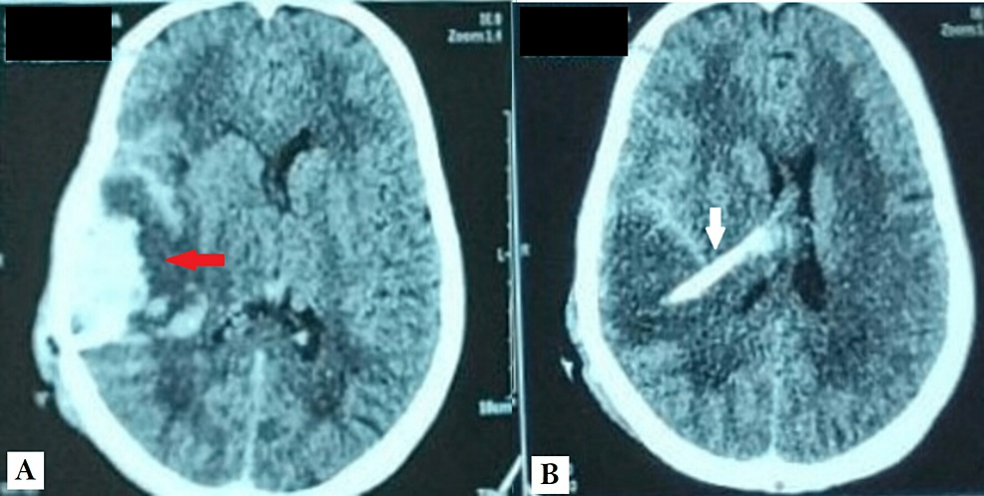
Control CT scan of the brain performed on the second postoperative day. (A) CT scan of the brain in axial planes showing a right temporoparietal hematoma (red arrow) on the trajectory of the VP shunt intracranial catheter. (B) CT scan of the brain in axial planes showing a right temporoparietal hematoma with the intracranial catheter (white arrow) in the right lateral ventricle. VP: ventriculoperitoneal.

On the second postoperative day, she presented with intense headaches and vomiting with preserved consciousness and a normal neurological examination. A control CT scan of the brain was ordered urgently, demonstrating a massive intra-cranial temporoparietal hematoma located in the trajectory of the VP shunt intracranial catheter (Figure 2). The headaches were managed with analgesics and corticosteroids (methylprednisolone bolus of 120 mg for a period of 48 hours) in addition to further administration of FFP (15-25 mL per kilogram of body weight), and the patient was closely monitored for any significant change in consciousness levels or neurological anomalies.

Three days after the incident, on postoperative day five, the patient showed a favorable evolution of her clinical symptoms as well as a significant volume reduction of the hematoma on a second control CT scan of the head. In light of the imaging results and clinical improvement, our patient was kept on medical treatment and monitoring and was discharged on postoperative day 10, with scheduled biological and imaging follow-up. The patient has been seen at her two months follow-up and showed clinical improvement with a normal physical examination.

## Discussion

FVD was first identified by Paul Owren in 1943 [1,10]. Its prevalence is estimated to be one in one million [2,9,11]. The mutational spectrum of FVD is extremely broad and variable, with the majority of patients having one or more rare mutations [12]. This results in diverse clinical presentations, ranging from simple mucosal bleeding to intracranial or digestive bleeding [1]. FVD can be categorized according to the factor V percentage in the plasma into three categories: mild, moderate, and severe [5]. In the presented case, the factor V percentage was 12%, corresponding to a mild form.

According to our patient’s history, she gave birth twice and underwent a celioscopic cholecystectomy with no significant bleeding complications. Taking into consideration the patient's current symptoms, the imaging findings of evolving triventricular hydrocephalus, as well as the previously mentioned history, a surgical treatment course consisting of a VP shunt was proposed despite the underlying risk.

Due to the rarity of these disorders and the scant literature available on the pre-operative preparations required for intracranial surgical procedures, the patient received the recommended volume of FFP according to the literature on other surgeries with high bleeding risks [9,13]. The calculated volume of FFP (15 to 25 mL of FFP per kilogram of body weight) is administered a short time before the procedure (30 min), aiming for a factor V level above 20% of normal [8,9,13]. Volume overload leading to acute lung injury or acute decompensation of pre-existing heart insufficiency is a significant danger, and these patients' volume status needs to be monitored during and after surgery [8]. Our patient did not present any of the described complications of FFP transfusion.

The surgical procedure was straightforward with no bleeding per operatively, and the immediate postoperative status of the patient was favorable. Nevertheless, we encountered postoperative intracranial bleeding, which attests to the lack of efficacy of the recommended dose in our patient despite a factor V level of 25% after transfusion. We managed this complication by surveillance and administration of FFP and symptomatic treatment. Fortunately, midterm follow-up was simple and showed favorable evolution along with regression of the intracranial hematoma.

## Conclusions

Congenital FVD is a rare severe bleeding disorder. A severe quantitative FV deficiency can lead to significant spontaneous bleeding episodes that pose a serious risk to life. Intravenous FFP is recommended in pre-operative preparation to improve the FV rate and reduce the probability of bleeding. Nevertheless, it is still unclear how FFP should be used in cases of intracranial surgery, and despite the recommendation, the bleeding risk cannot be predicted and fully avoided solely with these measures.

We stress the importance of a preanesthetic evaluation and a full risk evaluation prior to surgery. This can only be obtained through multidisciplinary collaboration between surgeons, hematologists, and anesthesiologists. Furthermore, protocols to manage these disorders have to be well developed and studied to allow better management of these patients, especially when surgery is inescapable.

## References

[1] Ehtisham M, Shafiq MA, Shafique M, Mumtaz H, Shahzad MN (2021). Owren's disease: a rare deficiency. Cureus.

[2] Yoneoka Y, Ozawa T, Saitoh A, Arai H (1999). Emergency evacuation of expanding intracerebral haemorrhage in parahaemophilia (coagulation factor V deficiency). Acta Neurochir (Wien).

[3] Mannucci PM, Duga S, Peyvandi F (2004). Recessively inherited coagulation disorders. Blood.

[4] Park YH, Lim JH, Yi HG, Lee MH, Kim CS (2016). Factor V deficiency in Korean patients: clinical and laboratory features, treatment, and outcome. J Korean Med Sci.

[5] Subramanian H, Kar R, Charles D, Babu H, Ambika P, Dutta TK (2017). A confounding case of inherited factor V deficiency complicated by inhibitors at first presentation. Transfus Med Hemother.

[6] Seeler RA (1972). Parahemophilia: factor V deficiency. Med Clin North Am.

[7] Huang JN, Koerper MA (2008). Factor V deficiency: a concise review. Haemophilia.

[8] Meidert AS, Kinzinger J, Möhnle P (2019). Perioperative management of a patient with severe factor V deficiency presenting with chronic subdural hematoma: a clinical report. World Neurosurg.

[9] Peyvandi F, Menegatti M (2016). Treatment of rare factor deficiencies in 2016. Hematology.

[10] Bernal S, Pelaez I, Alias L (2021). High mutational heterogeneity, and new mutations in the human coagulation factor V gene. Future perspectives for factor V deficiency using recombinant and advanced therapies. Int J Mol Sci.

[11] Acharya S, Panda S, Biswal S (2022). Primary hydatid cyst of neck, a rare case report. Indian J Otolaryngol Head Neck Surg.

[12] Thalji N, Camire RM (2013). Parahemophilia: new insights into factor v deficiency. Semin Thromb Hemost.

[13] Bello A, Salazar E, Heyne K, Varon J (2019). Aortic valve replacement in severe factor V deficiency and inhibitor: diagnostic and management challenges. Cureus.

